# Development and evaluation of e‐learning materials for dental hygiene students in six schools: Using smartphones to learn dental treatment procedures

**DOI:** 10.1111/idh.12452

**Published:** 2020-07-17

**Authors:** Akane Takenouchi, Etsuyo Otani, Masayo Sunaga, Takako Toyama, Hiromi Uehara, Kyoko Akiyama, Takae Kawashima, Kanade Ito, Hiromi Izuno, Atsuhiro Kinoshita

**Affiliations:** ^1^ Department of Educational Media Development Graduate School of Medical and Dental Sciences Tokyo Medical and Dental University Tokyo Japan; ^2^ Shinjuku Medical Career College Tokyo Japan; ^3^ Curricular Management Division Institute of Education Tokyo Medical and Dental University Tokyo Japan; ^4^ Department of Oral Health Sciences Faculty of Health Sciences Osaka Dental University Osaka Japan; ^5^ Department of Oral Health Kobe Tokiwa Junior College Hyogo Japan; ^6^ Department of Health Sciences School of Health and Social Services Saitama Prefectural University Saitama Japan; ^7^ Department of Oral Care for Systemic Health Support Health Sciences and Biomedical Engineering Graduate School of Medical and Dental Sciences Tokyo Medical and Dental University Tokyo Japan; ^8^ Department of Oral Health Sciences Faculty of Nursing and Health Care BAIKA Women's University Osaka Japan

**Keywords:** dental hygiene education, dental treatments, e‐learning, four‐handed dentistry, randomized controlled trial, smartphones

## Abstract

**Objective:**

The purpose of this study was to develop and evaluate the effectiveness of using e‐learning on smartphones to provide dental hygiene education on dental treatment procedures.

**Methods:**

This was a randomized controlled trial. Two‐hundred ninety‐three dental hygiene students in four universities, one junior college and one technical school took a preliminary examination, and based on the results, participants in each school were randomly divided into two groups, a test or control group. Both groups took pre‐ and post‐examinations at a 4‐week interval. The test groups learned dental treatment procedures and four‐handed techniques with interactive learning materials using smartphones. The learning materials allowed them to watch videos of dental treatments. The control groups were not provided any learning material.

**Results:**

Results of all schools combined showed that the changes from pre‐ to post‐examination scores in the test groups were significantly higher than those of the control groups (*p* < .05). Post‐examination scores were significantly higher than pre‐examination scores in the test groups in all schools (*p*  < .05). Also, post‐examination scores of the test groups were significantly higher than those of the control groups (*p* < .05). The changes from pre‐ to post‐examination scores in the test groups of two schools were significantly higher than those of control groups (*p* < .05). Post‐examination scores of the control groups in two schools were significantly higher than pre‐examination scores (*p* < .05).

**Conclusions:**

Learning dental treatment procedures through e‐learning on a smartphone was effective in developing participants’ understanding of dental treatment procedures and four‐handed techniques.

## 1 INTRODUCTION

In Japan, there are 123,831 employed dental hygienists and 90.6% of them work at private dental clinics. Japanese dental hygienists have three main areas of focus: prevention, patient education, and dental hygiene practice and dental assistance.^1^, ^2^ Therefore, all dental hygiene students have to learn treatments for prevention, techniques for patient education, and dental treatment procedures to complete a professional dental hygienist course. However, it is difficult for many students to understand the dental treatment procedures performed by dentists before observing actual dental treatments in clinical practice. Previous attempts administered questionnaires to supervisors of clinical practice reported that supervisors answered that most of dental hygiene students should learn dental assisting more before starting clinical practice.^3^ Even though dental hygienists do not perform the same treatments as dentists, they must learn four‐handed techniques to assist dentists and ensure smooth treatment. Nevertheless, classes on four‐handed techniques on dental treatments are limited since dental hygiene students are required to learn other professional subjects, including dental hygiene practice and oral care for elderly people.^4^, ^5^, ^6^ Learning materials that enable students to imagine dental treatment procedures and to develop their understanding without increasing the number of classes they must take are required.

Information and communication technology and electronic learning systems (e‐learning) have been gradually introduced in dental education, and positive effects have been identified.^7^ Most e‐learning materials can only be accessed on a personal computer (PC).^8^, ^9^, ^10^ However, some studies have shown that young people prefer to use their smartphones to access the Internet,^11^ which means that they use smartphones as information devices more than PCs. In fact, 99.4% of Japanese adults in their 20s possess a smartphone,^12^ and this rate is also increasing in other many countries.^13^, ^14^ One study found that dental students follow the same trend of smartphone use.^15^ Moreover, previous research showed that learning materials that can be accessed on mobile devices were effective in increasing students’ motivation for learning.^16^, ^17^ Therefore, development of e‐learning materials that can be accessed on smartphones is needed. Previous research showed that watching videos, which is considered one form of information and communication technology, is positively evaluated by dental hygiene students.^3^, ^18^ However, there have been few studies on the effectiveness of watching videos of dental treatments on dental hygiene students’ learning and understanding of four‐handed dental treatments. Therefore, the purposes of this study were to develop e‐learning materials for smartphones that allow dental hygiene students to watch videos of dental treatments and to evaluate their effectiveness.

## 2 STUDY POPULATION AND METHODOLOGY

The study protocol was approved by the Ethical Committee of Tokyo Medical and Dental University (Approval No. D2018‐082).

### 2.1 Determination of sample size

This study was conducted in four undergraduate universities, one junior college and one technical school, all of which consented to participate in this study. All participants took a preliminary examination and the examination score was set as the primary outcome because it was an objective measure. The sample size of each school was determined by power analysis. Mean intra‐group differences in examination scores of 5≧ with a standard deviation of 5 would require 17 participants per group to detect an effect with alpha = 0.05 and power = 0.8 (Statistical Discovery, SAS, Cary, NC, USA).

### 2.2 Participants

This study focused on dental hygiene students who had not yet begun clinical practice. Third‐year university students, second‐year junior college and technical school students were targeted for participation since university students start clinical practice in their third year, and junior college and technical school students start in their second year. Eighteen dental hygiene schools including universities, junior colleges and technical schools, in which educators teach whom authors could get in touch, were asked to participate in the study. And then, 6 schools including 4 universities, 1 junior college and 1 technical school accepted. Educators of these schools agreed that it is difficult for their students to completely understand dental treatment procedures and dental assisting techniques before starting clinical practice. All of 6 schools did not utilize information and communication technology when they taught dental treatment procedures. The numbers of participants in each school, labelled A to F, were as follows: A = 38, B = 20, C = 68, D = 28, E = 61, and F = 78. The total number of participants in this study was 293 (mean age ± standard deviation: 20.3 ± 2.0 years old). The trial was registered in the UMIN clinical trials registry (ID: UMIN000035544). Informed consent was obtained from all participants.

### 2.3 Learning materials

**Figure 1:**
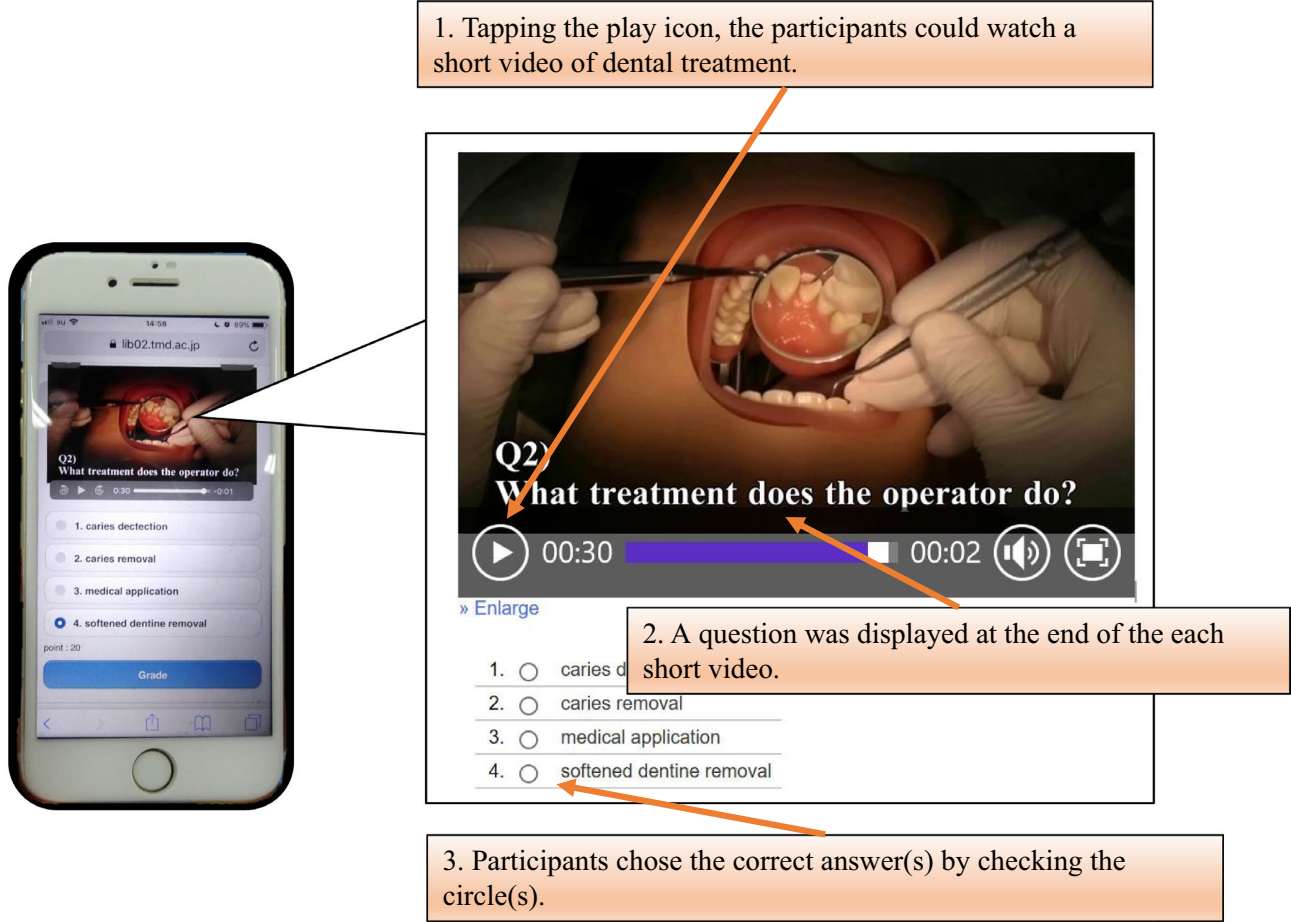
A screenshot of the learning materials [Colour figure can be viewed at wileyonlinelibrary.com]

Learning materials to acquire knowledge of dental treatment procedures that could be accessed on any type of information device were created by five educators who teach at a dental hygiene school or university. The materials were composed of five subtopics: resin restoration, inlay restoration, crown restoration, root canal treatment and fillings, and tooth extraction, which dental hygiene students should learn first before starting clinical practice.^3^ Participants utilized the learning materials in three steps (Figure 1): (a) participants tapped the play button and watched a short video, (b) a multiple choice question asking what instruments should be used or what the operator should do was asked at the end of each video, (c) participants chose the answer. Participants answered five questions for each subtopic. After answering the questions, they watched a series of videos describing each treatment procedure. These learning materials were delivered via the Learning Management System (WebClass^®^; Data Pacific (Japan) Ltd.), and the participant's access and use of the materials was recorded. The duration that participants spent watching videos with questions and descriptions by subtopic were as follows: resin restoration: 3.3 and 3.6 minutes; inlay restoration: 6.3 and 6.6 minutes; root canal treatment and fillings: 7.5 and 7.4 minutes; crown restoration: 4.8 and 6.6 minutes; and tooth extraction: 5.0 and 6.0 minutes. These learning materials were checked by specialists of each treatment, and modifications were conducted as needed.

### 2.4 Preliminary examination

The results of this examination were used for grouping of participants. The maximum examination score was 40. There were 20 multiple choice questions (2 points each). These questions asked about the procedures of each dental treatment, which were extracted from the past Dental Hygienist's National Examinations.

### 2.5 Pre‐ and post‐examinations

**Figure 2:**
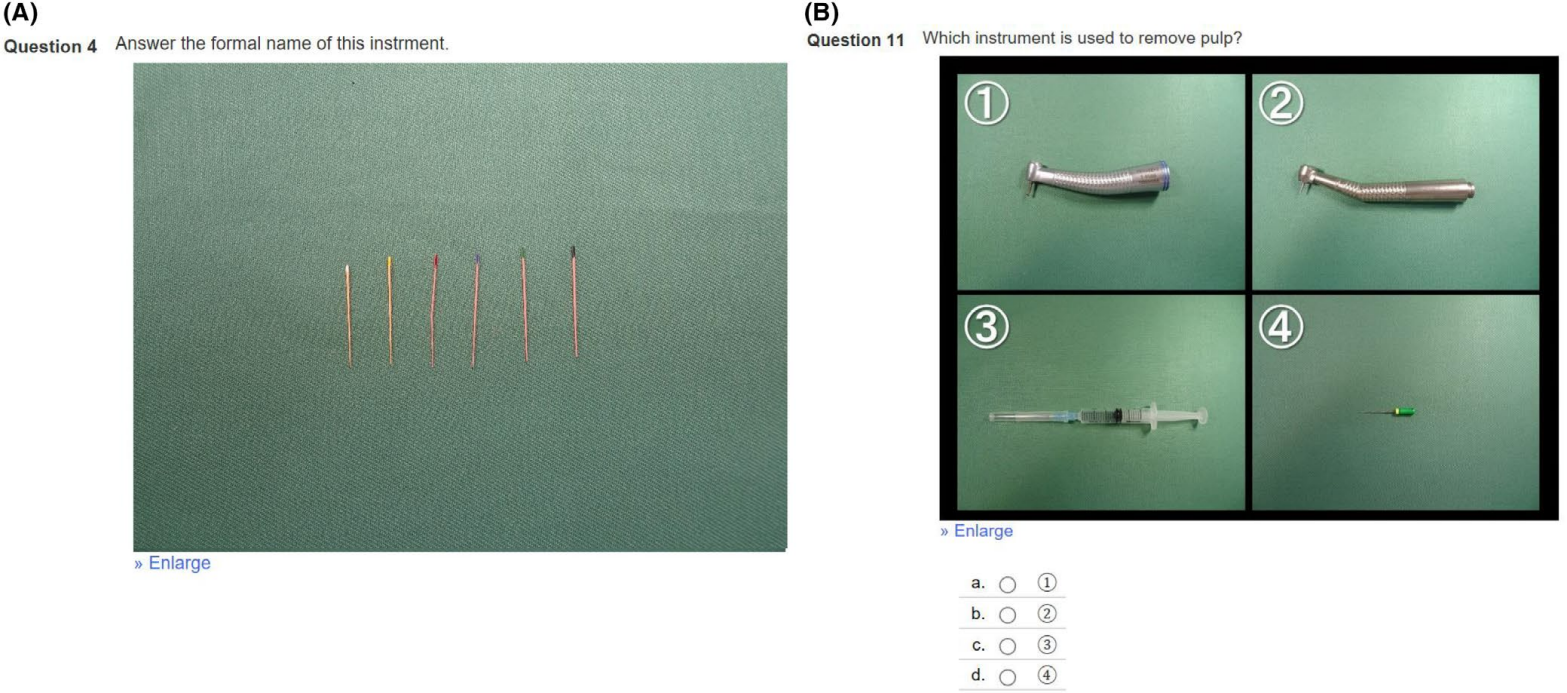
Screenshots of pre‐ and post‐examinations. A. Description question and B. multiple choice question [Colour figure can be viewed at wileyonlinelibrary.com]

The maximum examination score was 50. There were 15 multiple choice questions (2 points each), five multiple choice questions (3 points for each) and five description questions (1 point for each). Multiple choice questions about the procedures of each dental treatment were asked. Description questions about the names of instruments were also asked. A blinded examiner marked the participants’ responses to the description questions (see Figure 2).

### 2.6 Randomization

The participants at each school were systematically and randomly divided into two groups (a test group and a control group) according to the results of the preliminary examination and their experience as a part‐time dental assistant. Systematic randomization was conducted by listing the participants in descending order of preliminary examination scores in Microsoft Excel^®^ sheet. The each pair of participants from the top of the list were systematically and randomly allocated into either the test or control group. Randomization of each participant was generated by computer at the same time until the average preliminary examination scores and the number of students who with part‐time job experience as dental assistant roughly equal. There was no significant difference in preliminary examination scores between the two groups. In addition, the number of students who with part‐time job experience as a dental assistant was roughly equal between the group (see Table 1a and b).

**TABLE 1:** Examination scores

School	Number of participants	Number of participants with experience working as a dental assistant (%)	Number of participants without experience working as a dental assistant (%)	Preliminary examination score (Max points: 40)			Pre‐examination score (Max points: 49)			Post‐examination score (Max points: 49)			Change from pre‐ to post‐examination score		
				First quartile	Second quartile	Third quartile	First quartile	Second quartile	Third quartile	First quartile	Second quartile	Third quartile	First quartile	Second quartile	Third quartile
(a) Examination scores of the test groups															
A	18	4 (22%)	14 (78%)	19	24	28	13	17.5	21.8	15.5	22.5	29	0	5	8.8
B	10	2 (20%)	8 (80%)	18	22	26	12.5	15.5	22.3	14	23	30	0	3.5	12.5
C	34	11 (32%)	23 (68%)	14	17	20.5	9.8	14	16.3	16.5	20	27.3	3.5	7.5	13
D	14	5 (36%)	9 (64%)	6	8	8	10.3	15.5	20.3	14.5	22	26.3	0.8	4	8.5
E	31	7 (23%)	24 (77%)	10	12	18	8	11	17	10	15	20	‐3	3	8
F	38	9 (24%)	28 (74%)	10	16	20	10	14	18.3	11	15	23.3	‐0.3	2	5.3
(b) Examination scores of the control groups															
A	20	5 (25%)	15 (75%)	16	21	28	14.5	19	30.3	15	22	30.8	‐2.8	4	6
B	10	4 (40%)	6 (60%)	18	22	26	14.5	17.5	22.3	16.5	21.5	24.8	‐1.3	2	5
C	34	9 (26%)	25 (74%)	14	17	20.5	8.8	13	18.8	11	15	23.3	‐1.3	3	8
D	14	6 (43%)	8 (57%)	5.5	8	12	11.8	16.5	20	14	18.5	25.5	‐0.3	3	5.3
E	30	5 (17%)	24 (80%)	10	12	16.5	7	12	14	11	13.5	17.5	‐1	5	7
F	40	11 (28%)	27 (68%)	12	16	19.5	9	12.5	16	9	12	18	‐3	0	4
(c) Examination scores of the test groups in all schools combined															
All schools	145	36 (25%)	108 (74%)	10	16	20	10	14	19	13	18	25	0	4	8.5
(d) Examination scores of the control groups in all schools combined															
All schools	148	42 (28%)	103 (70%)	12	16	20	9.3	14	18	11	15	22	‐2	2	6

Abbreviation: SD, standard deviation.

### 2.7 Experimental design

**Figure 3:**
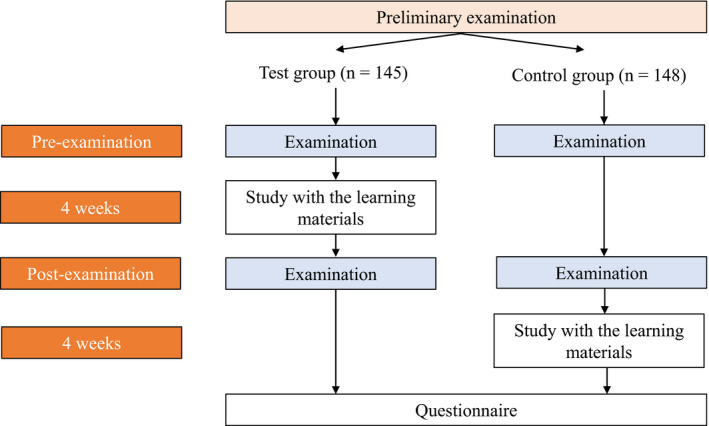
Flow chart which describes how the participants studied with the learning materials [Colour figure can be viewed at wileyonlinelibrary.com]

Both groups took pre‐ and post‐examinations with a 4‐week interval. The test group learned four‐handed dental treatment procedures using a smartphone. The control group was not given any learning materials. After the post‐examination, we provided the control group participants access to the learning materials to allow the same learning opportunity as was provided the test group participants. All participants had already finished the lectures related to the dental treatment procedures presented in the learning materials; therefore, the learning materials were used for review. All participants completed a questionnaire related to the learning materials after finishing the post‐examination. The questionnaire was composed of nine questions, including 7 Likert scale questions (see Figure 3) and the following 2 free response description questions: “Please describe the positive points of these learning materials”, and “Please describe how the learning materials should be improved.”

### 2.8 Statistical analysis

A non‐parametric test was used since pre‐examination scores showed a non‐normal distribution (Shapiro‐Wilk test). The Mann‐Whitney *U* test was used for inter‐group analysis of changes between pre‐ and post‐examination scores. The Wilcoxon signed rank test was used to evaluate changes between pre‐ and post‐examination scores between groups. Statistical analyses were conducted using JMP version 11 for Windows (Statistical Discovery).

## 3 RESULTS

The total average learning activity time (hours) and the completion rate of test groups in each school were as follows: A = 1.7 ± 0.8, 89%; B = 2.0 ± 0.9, 93%; C = 2.8 ± 1.3, 96%; D = 2.3 ± 1.3, 95%; E = 1.4 ± 0.8, 75%; and F = 1.7 ± 0.9, 85%. Watching less than 95% of the duration of each video was treated as material that was not learned. As shown in Table 2, the discrimination index of question 2 was 0.04, and therefore, it was considered an inappropriate question and was removed. Finally, the maximum score for both the pre‐ and post‐examinations was 49.

**TABLE 2:** Rate of correct answers and discrimination index of each question in the post‐examination

Question number	topic	Question type (point value)	Correct answer rate	Discrimination index
1	inlay restoration	Description questions (1 point for each)	0.65	0.32
2	resin restoration		0.13	0.04
3	crown restoration		0.09	0.14
4	root canal fillings		0.11	0.27
5	tooth extraction		0.05	0.15
6	resin restoration	Multiple choice questions (2 points for each)	0.52	0.22
7	resin restoration		0.26	0.51
8	inlay restoration		0.53	0.48
9	inlay restoration		0.28	0.47
10	root canal treatment		0.45	0.29
11	root canal treatment		0.53	0.62
12	root canal fillings		0.39	0.47
13	root canal fillings		0.39	0.67
14	crown restoration		0.43	0.40
15	crown restoration		0.41	0.60
16	crown restoration		0.41	0.60
17	tooth extraction		0.44	0.33
18	tooth extraction		0.49	0.45
19	tooth extraction		0.46	0.36
20	tooth extraction		0.21	0.40
21	resin restoration	Multiple choice questions (3 points for each)	0.34	0.29
22	inlay restoration		0.15	0.21
23	crown restoration		0.31	0.55
24	root canal treatment		0.36	0.29
25	tooth extraction		0.45	0.73

### 3.1 Examination scores

The post‐examination scores of the test groups were significantly higher than the pre‐examination scores in all schools (A: *p*  = .0315; B: *p* = .0234; C: *p* = .0001; D: *p* = .0029; E: *p*  = .0456; F: *p* = .0006). Post‐examination scores were significantly higher than pre‐examination scores in both groups (test group: *p* < .0001; control group: *p*  < .0001). Combined data of all school showed that the changes from pre‐ to post‐examination scores of the test groups were significantly higher than those of the control groups (*p* = .002). The changes from pre‐ to post‐examination scores in the test groups in schools C and F were significantly higher than those of the control groups in those schools (C: *p* = .002, F: *p* = .0336). The post‐examination scores of the control groups in schools C and E were significantly higher than the pre‐examination scores of the control groups in those schools (C: *p* = .022, E: *p*  = .0176). The post‐examination scores of the control groups schools A and B were higher than the pre‐examination scores of the control groups in those schools; however, the difference was not significant (A: *p*  = .056, B: *p* = .0977; see Tables 1 and 3).

**TABLE 3:** *p*‐values of intra‐ and inter‐group differences

School	Intra‐group differences		Inter‐group differences in changes from pre‐ to post‐examination
	Test group	Control group	
A	0.0315	0.056	NS
B	0.0234	0.0977	NS
C	0.0001	0.022	0.002
D	0.0029	NS	NS
E	0.0456	0.0176	NS
F	0.0006	NS	0.0336
All schools	<0.0001	<0.0001	0.002

Abbreviation: NS, not significant.

There were no significant differences in the changes from pre‐ to post‐examination scores by experience working as a part‐time dental assistant.

### 3.2 Questionnaire

**Figure 4:**
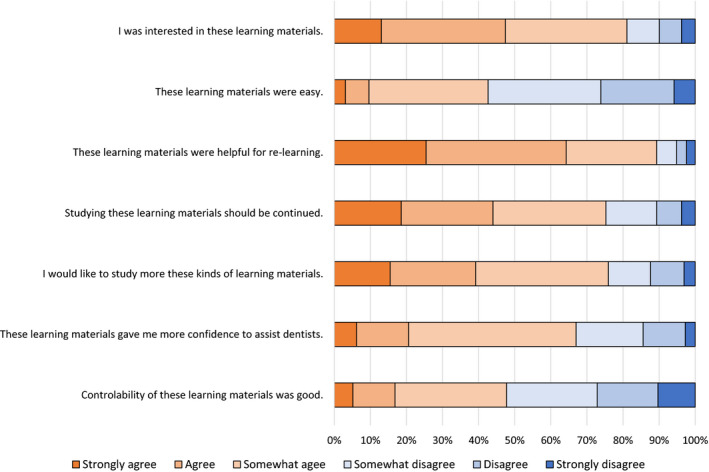
Results of questionnaire [Colour figure can be viewed at wileyonlinelibrary.com]

More than 80% of the participants answered positively to the questions, “These learning materials were helpful for reviewing,” and “I was interested in these learning materials.” About 50% of the participants answered positively to “These learning materials were easy to use,” and “These learning material content was easy.” Seventy‐three participants indicated that the “Videos were easy to understand,” and 37 participants indicated that “Audio explanations were easy to understand” as positive points. Sixty participants indicated that the “Videos of these learning materials often froze” as a point to be improved (see Figure 4).

## 4 DISCUSSION

Japanese dental hygiene students are required to understand dental treatment procedures and four‐handed techniques sufficiently to assist dentists and to ensure smooth dental treatment, even though they do not perform those dental treatments themselves. In this randomized controlled study, e‐learning materials that could be accessed using smartphones were developed and their effectiveness was evaluated objectively. The results of this study showed that learning dental treatment procedures using the developed learning materials was effective in significantly enhancing participants’ understanding of dental treatment procedures. We observed three main findings from our data.

First, e‐learning materials that allowed students to watch videos of dental treatments were effective in presenting images of real dental treatment procedures. Watching videos enabled participants to understand how an operator performs the treatment and uses instruments even though they have never watched dental treatments in person. Actually, some previous studies concluded that in addition to demonstrations, procedural videos can improve learners’ professional skills. ^19^, ^20^, ^21^ Some dental hygiene students could have difficulty imagining the details of dental treatments from verbal explanations or written materials with photographs; however, the procedure could be easier to follow by watching videos, which also allows them to see the operator's actual motions while performing four‐handed dental treatment procedures. Also, students prefer watching videos for learning to traditional learning method.^22^, ^23^ Furthermore, the introduction of videos in dental hygiene education can lead to more uniform education and help lessen educators’ workload since the time needed to perform demonstrations could be reduced. Demonstrations differ depending on the operator, which means that the contents and quality of lectures are not uniform. If educators could reduce the time spent preparing for and conducting demonstrations, they could gain creative time to improve their teaching and coaching skills and enhance the quality of their lectures. In addition, e‐learning materials that allow learners to watch videos will be useful tools to train not only dental hygiene students, but also dental hygienists who return to work after an absence. Japan currently has a shortage of working dental hygienists and Japan Dental Hygienists’ Association work to support dental hygienists’ reinstatement after absences.^24^ Increasing the number of working dental hygienists would lead to better promotion of oral health among the general population. More e‐learning materials that utilize videos should be developed in dental hygiene education.

Second, more e‐learning materials that can be accessed on smartphones should be developed and introduced in dental hygiene education. The results of the present questionnaire showed that most participants were interested in the learning materials since they could learn by using their own smartphone, which is the most user‐friendly device for them. In fact, young people like watching videos on YouTube and prefer using smartphones to PCs.^25^ If these learning materials were accessible only on a PC, the questionnaire responses would have been different. Furthermore, learning materials that can be accessed on smartphones could be introduced in all kinds of dental hygiene schools. In Japan, there are 168 dental hygiene schools, which include universities, junior colleges and technical schools, and they have different facilities and programs (eg some schools do not have PC rooms for students). However, in today's technological society, most students and educators have their own smartphones. Therefore, educators are able to introduce such learning materials easily, thereby providing equal learning opportunities to all students. However, control of the learning materials developed in this study should be improved since half of the participants were dissatisfied because the videos did not play quickly and smoothly. It is important to keep the duration of each video short so that they can stream smoothly on any device. It is also essential for educators to provide good Internet access for students to learn with e‐learning materials without any inconvenience.

Third, four factors are expected to have significantly developed participants’ understanding of the learning materials in the present study, but additional studies should be conducted to confirm these findings. First, watching videos of dental treatments enabled participants to imagine real dental treatments. Second, interactive learning by answering questions to confirm understanding and reviewing explanations enhanced participants’ comprehension of the material. Third, using smartphones increased participants’ motivation to learn and improved their understanding.^26^ Fourth, audio explanations in the videos enabled participants to understand how to use each instrument and what the operators were doing in the video.

As for the limitations of this study, only the test group used the learning materials during the 4‐week study period, while the control group was not allocated any learning materials, which means that the learning time of only the test group was increased. This could be one of the factors that improved post‐examination scores among test group participants. In a future study, both the test and control groups should be allocated materials on the same topics using different learning methods to more clearly identify the effectiveness of the learning materials. For instance, the test group could learn about dental treatment procedures using e‐learning materials, while the control group could learn using traditional handouts. Identifying the factors that highly impact students’ understanding and motivation to learn will lead to the development of high‐quality learning materials and heighten the quality of dental hygiene education. Also, it was another limitation that the gain of 8% in examination score of test group was not outstanding improvement. If the learning duration was prolonged or the contents of learning materials were improved, higher changes would be found. However, it is difficult to understand dental treatment procedures and acquire dental assisting techniques completely before starting clinical practice. Then, gain of 8% in examination score after learning using smartphones by themselves was not too low. Furthermore, not only acquirement of knowledge but also skills should be evaluated in the future study to evaluate effectiveness of learning materials developed in this study.

## 5 CONCLUSION

In this randomized controlled study, e‐learning materials that could be accessed on smartphones were developed, and these materials allowed participants to learn dental treatment procedures over a 4‐week study period. The results of this study showed that learning dental treatment procedures with e‐learning materials was effective in significantly enhancing participants’ understanding of the materials.

## 6 CLINICAL RELEVANCE

Scientific rationale for study: Novel learning materials and methods in dental hygiene education should be developed since the demand for dental hygienists have recently increased.

Principal findings: Learning materials that could be accessed on smartphones and allowed participants to watch videos were effective in improving participants’ understanding of dental treatment procedures.

Practical implications: Dental hygiene educators should develop and introduce e‐learning materials to heighten the quality of dental hygiene education. Dental clinics and hospitals should also incorporate these learning materials in their training for newly employed dental hygienists.

## CONFLICT OF INTEREST

The authors have declared that there are no conflicts of interest to disclose in relation to the publishing of this research.

## AUTHOR CONTRIBUTIONS

Akane Takenouchi and Atsuhiro Kinoshita designed the study protocol and wrote manuscript. Etsuyo Otani generated randomization. Masayo Sunaga managed e‐learning management system. Takako Toyama, Hiromi Uehara, Kyoko Akiyama, Takae Kawashima, Kanade Ito and Hiromi Izuno supervised pre‐ and post‐examinations.
